# A Review of Natural Language Processing in Medical Education

**DOI:** 10.5811/westjem.2018.11.39725

**Published:** 2018-12-12

**Authors:** Michael Chary, Saumil Parikh, Alex F. Manini, Edward W. Boyer, Michael Radeos

**Affiliations:** * New York-Presbyterian/Queens, Department of Emergency Medicine, Flushing, New York; † Boston Children’s Hospital, Harvard Medical Toxicology, Boston, Massachusetts; ‡ Brigham and Women’s Hospital, Department of Emergency Medicine, Boston, Massachusetts; § Icahn School of Medicine at Mount Sinai, Elmhurst Hospital Center, Department of Emergency Medicine, New York, New York; ¶ Coney Island Hospital, Department of Emergency Medicine, Brooklyn, New York

## Abstract

Natural language processing (NLP) aims to program machines to interpret human language as humans do. It could quantify aspects of medical education that were previously amenable only to qualitative methods. The application of NLP to medical education has been accelerating over the past several years. This article has three aims. First, we introduce the reader to NLP. Second, we discuss the potential of NLP to help integrate FOAM (Free Open Access Medical Education) resources with more traditional curricular elements. Finally, we present the results of a systematic review. We identified 30 articles indexed by PubMed as relating to medical education and NLP, 14 of which were of sufficient quality to include in this review. We close by discussing potential future work using NLP to advance the field of medical education in emergency medicine.

## INTRODUCTION

We use the term natural language processing (NLP) to refer to the field that aims to enable computers to parse human language as humans do. NLP is not a single technique; rather, it is composed of many techniques grouped together by this common aim. Two examples of NLP at an individual level are International Business Machine’s Watson™ and Apple’s Siri®. For example, Watson used NLP to convert each question on *Jeopardy!* into a series of queries that it could ask its databases simultaneously.^1^ Siri uses NLP to translate speech into commands to navigate the iPhone® or search the Internet.^2^

NLP reformats text to make that text amenable for subsequent analysis with techniques from machine learning or artificial intelligence. That text may come from clinician documentation, billing documentation, transcripts of patient-provider or provider-provider interactions, or even social media discussions. It converts text into a textual data stream that may be paired with data streams from physiological monitors (cardiac monitors, pulse oximetry), wearables, or laboratory tests. NLP has been successful in scaling up some components of medical decision-making, developing tools for risk stratification,^3^ identifying postoperative complications after inpatient surgery from physician notes,^4^ and triaging patients by identifying syndromes.^5^

### A Primer on Natural Language Processing

An important use of NLP is to translate, or map, words or phrases onto concepts. We want the computer to look past the sequence of letters to the concept denoted. We do not parse *hypoxia* as merely a string of letters. Mapping from words or phrases to concepts involves: 1) breaking a sentence into tokens (tokenization); 2) lemmatizing each token (lemmatization); and 3) mapping each lemma (the standard form of a word) onto one or more concepts. Some applications of NLP only perform steps 1 and 2, analyzing lemmata instead of concepts. This is appropriate for a domain where there is no accepted mapping between lemmata and concepts, or where the mapping is very close to one-to-one.

### Tokenization

A *token* is a word or phrase that refers to one concept; for example, *cell* and *mast cell* are both tokens. A common algorithm for breaking a sentence into tokens, termed tokenization, is to break a phrase on spaces. Breaking on spaces converts the sentence *the quick fox jumped over the lazy dog* into the list of tokens [*the*, *quick*, *fox*, *jumped*, *over*, *the*, *lazy*, *dog*]. Breaking a string on spaces is inadequate for technical vocabulary where a token may involve multiple words, for example *mast cell* or *red blood cell*. Most modern programming languages, including C, R, Python, Ruby, Java, and Clojure have libraries or plugins that can tokenize English words.

### Lemmatization

The lemma of a word is the form of that word that would be found in a dictionary. Standardization, or (preferably) lemmatization, refers to the process of mapping a token, for example *red blood cells*, onto a lemma, here *erythrocyte*. Lemmatizing may also include standardizing spelling (e.g., mapping “tonight” and “tonite” both to “tonight”) and expanding abbreviations (e.g., mapping DOE to dyspnea on exertion). The word lemma is the linguistic term for the base form of a word. Most modern programming languages, including C, R, Python, Ruby, Java, and Clojure have libraries or plugins that can lemmatize English words.

The traditional order of NLP is first to tokenize and then lemmatize the text. It may be more productive to lemmatize, tokenize, and lemmatize texts that contain medical vocabulary. The first lemmatization maps all words or phrases to their dictionary form. Before tokenization, phrases from the text can be removed if they occur on a list. This provides a transparent way to identify in the text and move to a list of tokens phrases, such as *mast cell* or *red blood cell*, without having to enumerate all lexical (spelling) variants of each phrase. A similar approach can be used to create lists of words that are to be removed from the text and discarded. These words, termed stopwords, are words that are considered noise for the topic at hand. In our experience we’ve found that it is better to leave stopwords in, if possible. The most common words in the English language are stopwords. Leaving stopwords in provides an internal control for analysis methods that hinge on comparing the frequencies of tokens.

### Mapping a Lemma to a Concept

The mapping of a word to a concept is difficult. A word has many meanings and many words express the same meaning, a phenomenon termed polysemy. The mapping can change over time as the meaning or popularity of a word changes. The meaning of a word may depend on the speaker and context.

One successful and automated approach groups lemmata together based on their patterns of occurrence in a body of text (corpus). The underlying conceptual hypothesis is that lemmata whose patterns of occurrence are statistically significantly correlated are describing the same thing. The term *topic* is usually used instead of concept to denote that words found by statistical co-occurrence may not share as close a meaning as the phrase “referring to the same concept” implies. Although this approach is quick and not overwhelmed by large amounts of data, its conceptual hypothesis suffers from the same weakness, as do all approaches that attempt to infer meaning from the frequency of tokens or lemmata. The most frequent words may not be the most important words. While words such as *unremarkable* or *normal* are ubiquitous in clinical documentation, they are less informative than rarer phrases such as *absent lung sounds*.

### Latent Dirichlet Allocation

Latent Dirichlet allocation (LDA), also called topic modeling, expresses a piece of text as a weighted linear combination of topics, just as a generalized linear model expresses a dependent variable as a weighted linear combination of independent variables. All documents are composed by mixing the same topics. One document differs from another in the relative weight it gives to each topic. In LDA, topic denotes a group of words that occur together more often than would be expected by chance. The set of words [coronary, artery, disease], for example, could be a topic. LDA topics are correlated because they share words and so cannot be considered independent variables. This may make it difficult to include the results of topic modeling in multivariate regression models.

### How Natural Language Processing Could Help Medical Education in Emergency Medicine (EM)

NLP could help medical education in EM in the following ways:

1. By applying techniques used to analyze trainee documentation in other areas to analyze documentation in the emergency department
2. By applying NLP techniques to FOAM.

### Analyzing EM Documentation to Track Resident Performance

Graduate medical education in EM aims to produce emergency physicians. The assessment of medical knowledge occurs, traditionally, through standardized oral and written exams. NLP provides a way to infer the development of medical decision-making from the documentation that residents routinely generate. This evaluation occurs continuously, unobtrusively, and in the resident’s usual working environment.

**Figure 1:**
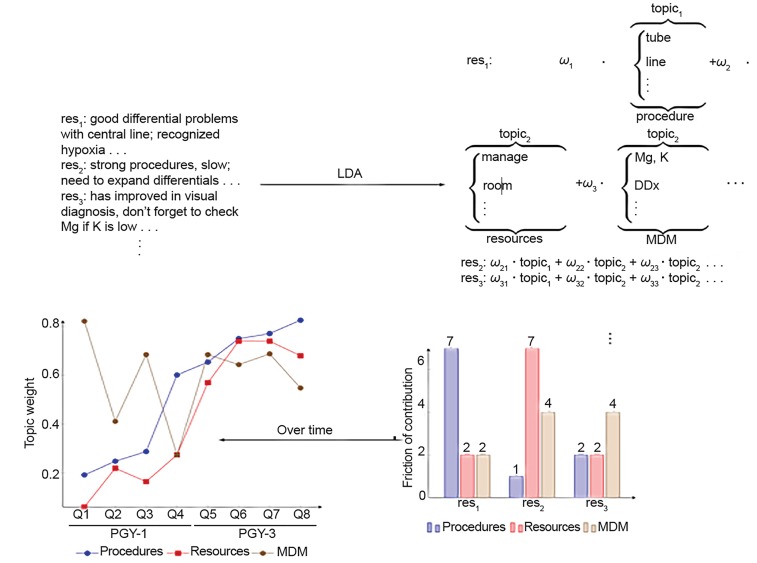
Hypothetical example of the use of natural language processing to quantify the evolution of resident medical decision-making as assessed by attending evaluations. [Schematic made by authors]. *PGY*; post graduate year; *Q*, quarter; *MDM*, medical decision making; *Mg*, magnesium; *K*, potassium; *DDx*, differential diagnosis; *ω*, topic weight; LDA, latent Dirichlet allocation.

Figure 1 is our schematic of how NLP could be used to compare three residents as they progress in training. The upper left corner shows sample inputs, which could be evaluations completed by attendings after a shift. Performing LDA on that text, after preprocessing, tokenizing, and lemmatizing, yields the topics in the upper right. A lemma can belong to more than one topic, although Figure 1 shows parts of topics with unique words for the sake of exposition. The labels for each topic (underbrace text) are generated by expert review, not the LDA algorithm. The manual review of topics provides a natural point for investigators to check the quality of their data and analysis. The topics are the same across all residents. The weights differ, as the subscripts indicate. One can track the value of these weights cross-sectionally (lower right panel) or longitudinally (lower left panel). This tracking can be done automatically and continuously, allowing each resident to be compared with an ever-growing reference database.

### Free Open Access Medical Education (FOAM)

FOAM is an increasingly prominent source of asynchronous education materials.^6^ FOAM resources include websites, podcasts, or blog posts where those interested in emergency care discuss, comment, and provide access to content related to emergency care. Few FOAM resources are peer-reviewed. FOAM and social media provide a way for residents to engage with the dissemination and incorporation of (new) knowledge into EM. The structure, scale, and variable quality of FOAM, however, make these resources difficult to include in residency training. NLP could provide structure to FOAM and social media, making it easier to incorporate these resources into residency curricula. Manual curation of parts of FOAM risks missing resources and is time-consuming.

NLP could help residents prioritize FOAM resources in the following way: A group of experts constructs topics it agrees is essential for any FOAM article to have; we would then use topic modeling to identify which FOAM resources have enough of these topics. An alternative method is to determine which topics are present in FOAM resources to see whether there is any intrinsic ordering to FOAM resources.

NLP could help organize FOAM by identifying which topics were most prevalent. A cross-sectional analysis of the relative prevalence of topics could be informative in identifying areas relatively lacking in discussion. A manual curation of those topics could identify lemmata that were markers of quality. A subsequent algorithm could use these markers of quality to automatically rate each website, in effect scaling up the efforts by Academic Life in Emergency Medicine (ALiEM), which currently rely on a panel of experts to review each blog post.^7^,^8^ In addition, NLP could quickly reassess resources whose content has changed.

## METHODS

**Figure 2:**
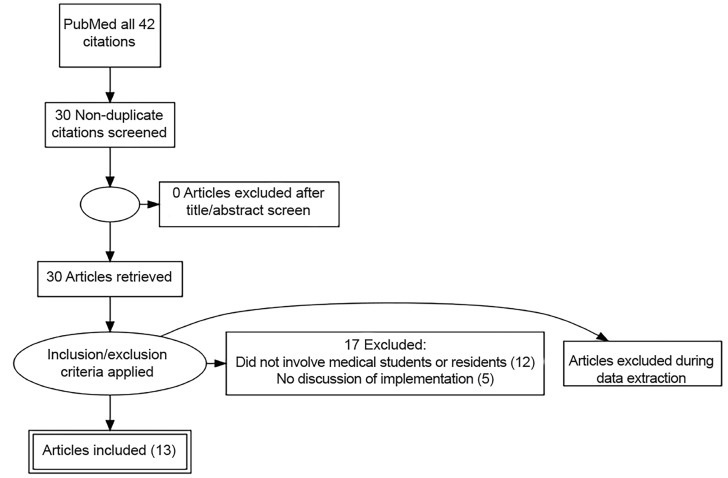
Preferred reporting items for systematic reviews and meta-analyses (PRISM-A) style flowchart detailing extraction, screening, and inclusion of articles.

To gauge how researchers are using NLP to evaluate medical students or residents, we searched PubMed for all English-language full-text case reports, clinical trials, or original research articles that contained the text “natural language processing in medical education.” Our search identified 30 articles. We divided the studies into five categories: patient simulation, evaluation of documentation, tracking clinical exposure, question banks, and “not related.” From those 30 articles authors MC and AM identified, through manual curation, 13 that described the use of NLP in medical education. Figure 2 summarizes our acquisition of data in Preferred Reporting Items for Systematic Reviews and Meta-Analyses (PRISM-A) format. Table 1 describes the 17 studies excluded from further analysis because they did not involve the analysis with NLP of resident or medical student textual output. Table 2 lists the 13 studies that were analyzed. The rest of this article discusses only those manuscripts related to the evaluation of documentation.

## RESULTS

### Evaluation of Documentation

Zhang et al. demonstrated that latent Dirichlet allocation could be used to quantify the degree to which attending feedback to a resident evaluated that resident from the perspective of each of the Accreditation Council for Graduate Medical Education (ACGME) milestones and the degree to which the feedback was positive or negative.^9^ An improved methodology could be used to track this sentiment for each milestone over time to automatically identify residents with a change in resident sentiment. The improvements would be to use lemmata instead of words, allow words to be associated with more than one milestone, and to validate the evaluations of residents the algorithm produces against the actual evaluations of those residents. Such an automated curation of attending evaluations could provide objective context as to whether one incident was isolated or one in a long train of similar incidents. Because software identifies the problem, it removes the question of personal bias and may help to focus the discussion more about the issue than who identified the issue.

Denny et al. used NLP to evaluate the ability of third-year medical students to develop a full differential for altered mental status in the elderly patient and discuss advance directives.^10^ In that study a computer program analyzed the notes each student wrote every day to identify whether the medical student had participated in a goals-of-care discussion if the patient was over 65, and the patient was being evaluated for altered mental status. If the patient was being evaluated for altered mental status, the algorithm also assessed whether the medical student had generated a comprehensive differential.

This study mapped text to medical concepts by tokenizing the student notes, normalizing those tokens to lemmata, and mapping each lemma from each note onto Unified Medical Language System (UMLS) tags. (Lemma refers to the standard form of a word; see “An Introduction to Natural Language Processing” below.) The authors used the same system to assess the prevalence of key concepts, as defined by the American Association of Medical Colleges, a medical student must-see during his or her medical clerkship.^12^,^13^ The authors used the UMLS Metathesaurus, a graph of semantic relationships between words, to map a lemma to the concepts it likely represents. Each concept is represented by a basket of lemmata.^14^ The algorithm marks a student note as containing that concept if that note contained a lemma. The software uses context clues to choose which lemma-concept mapping is the most likely. This study provides an example of how NLP may also improve documentation by medical students by providing an “enhanced spell-checker” while providing real-time feedback that has educational value. The Center for Medicaid Services allows physicians to document the review of systems, past family history, and past social history documented by medical students.^11^

Zhang et al. used latent Dirichlet allocation, also called topic modeling, to quantify how much of each ACGME milestone was reflected in free-text evaluations by attending internists on medical residents and whether the reflection was positive or negative.^9^ The authors used topic modeling to identify clusters of thematically-related words in attending free-text evaluations. They then manually inspected each cluster, labeling each cluster as indicative of only one ACGME milestone (e.g., problem-based learning and instruction, or professionalism, or systems-based practice). The authors then calculated the relative prevalence of each cluster/milestone in each note and sentiment, also called emotional valence, associated with those prevalences. The authors, however, did not assess whether the calculated prevalences or the cluster assignments agreed with attending impression. The requirement that each word belong to only one cluster simplifies the calculation of prevalence, but may be an oversimplification. A word can have multiple meanings. The ACGME milestones are not entirely separate domains. For example, some overlap is to be expected in words used to evaluate a resident in the domains of professionalism, patient-based care, and systems-based practice.

Da Silva and Dennick tokenized transcripts from problem-based learning sessions involving attending physicians and a group of first- or second-year medical students.^15^ The authors identified common medical words or phrases, such as hepatitis or red blood cell. Across three sessions, the authors demonstrated an increase in the interposition of subordinating conjunctions between tokens representing medical concepts – hepatitis (medical token) caused by (subordinating conjunction) alcohol (medical token). This suggests that participants were verbalizing, and perhaps integrating, more clinical reasoning as the sessions progressed.

## LIMITATIONS

The purpose of this article was to provide medical educators with an introduction to NLP and survey current applications of NLP that may be of interest to educators. A limitation ubiquitous in any survey of a field is publication bias. This article only considered published manuscripts, which may provide a biased representation of the scope and success of the field. Some researchers may release source code for software to online platforms such as GitHub or describe their research via social media.

## CONCLUSION

This article reviewed recent applications of natural language processing to medical education, introduced concepts from NLP used in those applications, and then suggested avenues for its application to medical education in EM. Incorporating NLP into residency education could help program directors better track the progression of their residents across quantitative and qualitative domains, automatically and continuously. Residents with diverse backgrounds, from the humanities to programming, and diverse interests, from international EM to informatics, could contribute to the development of NLP tools, the incorporation of existing NLP tools into the clinical workflow, or the inclusion of FOAM resources into residency education. NLP provides a way to represent clinical reasoning in a form that computers can understand, perhaps one day creating something that can access data at the speed of a computer and reason with the abstraction of an outstanding clinician.

## Figures and Tables

**Table 1:** Seventeen studies that were excluded from further analysis.

Citation	Title	Level of evidence	Reason excluded
Evaluation of documentation			
Madhavan et al. (2014). *J Grad Med Educ*. 2014;6(3):577–80. doi: 10.4300/JGME-D-13-00267.1. PMID: 26279789	Evaluation of Documentation Patterns of Trainees and Supervising Physicians Using Data Mining	2	Analyzes when trainees and attendings document, not what they document
Divita et al. (2017) *Stud Health Technol Inform*. 2017;245:356–60. doi: 10.3233/978-1-61499-830-3-356 PMID: 29295115	General Symptom Extraction from VA Electronic Medical Notes	4	Does not discuss medical education
Park et al. (2015) *AMIA Annu Symp Proc*. 2015:1024–33. eCollection 2015. PMID: 26958240	Homophily of Vocabulary Usage: Beneficial Effects of Vocabulary Similarity on Online Health Communities Participation	4	Does not involve medical students or residents
Park et al. (2015) *J Med Internet Res*. 2015;17(8):e212. doi: 10.2196/jmir.4612. PMID: 26323337	Automatically Detecting Failures in Natural Language Processing Tools for Online Community Text	4	Does not involve medical students or residents
Karmen et al. (2015) *Comput Methods Programs Biomed*. 2015;120(1):27–36. doi: 10.1016/j.cmpb.2015.03.008. PMID: 25891366	Screening Internet forum participants for depression symptoms by assembling and enhancing multiple NLP methods	3	Does not involve medical students or residents
Turner et al. (2015) *J Biomed Inform*. 2015;53:136–46. doi: 10.1016/j.jbi.2014.10.005. PMID: 25445922	Modeling workflow to design machine translation applications for public health practice	5	Does not involve medical students or residents
Turner et al. (2015) *Stud Health Technol Inform*. 2015;216:979. doi: 10.2196/publichealth.4779 PMID: 26262281	Machine assisted Translation of Health Materials to Chinese: An Initial Evaluation	4	Does not involve medical students or residents
Radiology			
Solti et al. (2009) *Proceedings (IEEE Int Conf Bioinformatics Biomed)*. 2009:314–19. doi: 10.1109/BIBMW.2009.5332081 PMID: 21152268	Automated Classification of Radiology Reports for Acute Lung Injury: Comparison of Keyword and Machine Learning Based Natural Language Processing Approaches	4	Does not involve medical students or residents
Hersh et al. (2001). *J Biomed Inform*. 2001;34(4):262–73. doi: 10.1006/jbin.2001.1025 PMID: 11977808	Selective automated indexing of findings and diagnoses in radiology reports	4	Does not involve medical students or residents
Overby et al. (2009) *BMC Bioinformatics*. 2009;10 Suppl 9:S8. doi: 10.1186/1471-2105-10-S9-S8. PMID: 19761578	The potential for automated question answering in the context of genomic medicine: An assessment of existing resources and properties of answers	5	Not a primary research article Does not involve medical students or residents Duplicate of prior article
Rosse and Mejino (2003). *J Biomed Inform*. 2003;36(6):478–500. doi: 10.1016/j.jbi.2003.11.007 PMID: 14759820	A reference ontology for biomedical informatics: the Foundational Model of Anatomy	5	Does not involve medical students or residents
Wehbe et al. (2003) *AMIA Annu Symp Proc*. 2003:1049. PMID: 14728552	Formative evaluation to guide early deployment of an online content management tool for medical curriculum	5	Does not involve medical students or residents Describes content development, but no implementation or evaluation
Distelhorst et al. (2003). *AMIA Annu Symp Proc*. 2003:200–4. PMID: 14728162	A prototype natural language interface to a large complex knowledge base, the Foundational Model of Anatomy	4	Does not involve medical students or residents, interface intended for “domain experts in anatomy”
Chu and Chan (1998). *Comput Biol Med*. 1998;28(5):459–72. doi: doi.org/10.1016/S0010-4825(98)00027-4 PMID: 9861505	Evolution of web site design: implications for medical education on the Internet	5	Not a primary research article
Séka et al. (1998). *Stud Health Technol Inform*. 1998;52 Pt 2:772–6. doi: 10.3233/978-1-60750-896-0-772 PMID: 10384566	A virtual university web system for a medical school	4	Describes content development, but no implementation or evaluation
Webhe and Spickard (2005). *AMIA Annu Symp Proc*. 2005:794–8. PMID: 16779149	How students and faculty interact with a searchable online database of the medical curriculum	3	Compares trainee and attending interaction with a previously created database Creation of database involved NLP
Patient simulation			
Persad et al. (2016). *Med Educ*. 2016;50(11):1162–63. doi: 10.1111/medu.13197. PMID: 27762013	A novel approach to virtual patient simulation using natural language processing	4	Structured abstract incorrectly marked as a manuscript
Oliven et al. (2011) *Stud Health Technol Inform*. 2011;169:233–7. doi: 10.3233/978-1-60750-806-9-233 PMID: 21893748	Implementation of a web-based interactive virtual patient case simulation as a training and assessment tool for medical students	4	No description of NLP techniques used

*doi*, digital object identifier; *PMID*, PubMed IDentifier; *VA*, Veterans Affairs; *NLP*, natural language processing.

**Table 2:** Studies included for further analysis.

Citation	Title	Level of evidence
Evaluation of documentation		
Denny et al. (2015). *J Biomed Inform*. 2015;56:292–9. doi: 10.1016/j.jbi.2015.06.004. PMID: 26070431	Using natural language processing to provide personalized learning opportunities from trainee clinical notes	3
Denny et al. (2010). *AMIA Annu Symp Proc*. 2010;2010:157–61. PMID: 21346960	Comparing content coverage in medical curriculum to trainee-authored clinical notes	3
Spickard et al. (2014). *Med Teach*. 2014;36(1):68–72. doi: 10.3109/0142159X.2013.849801. PMID: 24195470	Automatic scoring of medical students’ clinical notes to monitor learning in the workplace	
Zhang et al. (2012). *AMIA Annu Symp Proc*. 2012;2012:1459–68. PMID: 23304426	Automated assessment of medical training evaluation text	3
Da Silva, Dennick (2010). *Med Educ*. 2010;44(3):280–8. doi: 10.1111/j.1365-2923.2009.03575.x. PMID: 20444059	Corpus analysis of problem-based learning transcripts: an exploratory study	4
Tracking clinical exposure		
Denny et al. (2009). *J Biomed Inform*. 2009;42(5):781–9. doi: 10.1016/j.jbi.2009.02.004. PMID: 19236956	Tracking medical students’ clinical experiences using natural language processing	3
Chen et al. (2014). *AMIA Annu Symp Proc*. 2014;2014:375–84. eCollection 2014. PMID: 25954341	Automated Assessment of Medical Students’ Clinical Exposures according to AAMC Geriatric Competencies	3
Question banks		
Wedgwood (2005). *AMIA Annu Symp Proc*. 2005:1150. PMID: 16779436	MQAF: a medical question-answering framework	4

*PMID*, PubMed identification; *doi*; digital object identifier; *AAMC*, Association of American Medical Colleges.
